# Cultural Adaptation of the Portuguese Version of the “*Sniffin’ Sticks*” Smell Test: Reliability, Validity, and Normative Data

**DOI:** 10.1371/journal.pone.0148937

**Published:** 2016-02-10

**Authors:** João Carlos Ribeiro, João Simões, Filipe Silva, Eduardo D. Silva, Cornelia Hummel, Thomas Hummel, António Paiva

**Affiliations:** 1 Department of Otorhinolaryngology, Coimbra University Hospital (CHUC), Coimbra, Portugal; 2 Faculty of Medicine, University of Coimbra, Coimbra, Portugal; 3 Centre of Ophthalmology and Vision Sciences, IBILI, Faculty of Medicine, University of Coimbra, Coimbra, Portugal; 4 Department of Otorhinolaryngology, University of Dresden Medical School, Dresden, Germany; Duke University, UNITED STATES

## Abstract

The cross-cultural adaptation and validation of the Sniffin`Sticks test for the Portuguese population is described. Over 270 people participated in four experiments. In Experiment 1, 67 participants rated the familiarity of presented odors and seven descriptors of the original test were adapted to a Portuguese context. In Experiment 2, the Portuguese version of Sniffin`Sticks test was administered to 203 healthy participants. Older age, male gender and active smoking status were confirmed as confounding factors. The third experiment showed the validity of the Portuguese version of Sniffin`Sticks test in discriminating healthy controls from patients with olfactory dysfunction. In Experiment 4, the test-retest reliability for both the composite score (r_71_ = 0.86) and the identification test (r_71_ = 0.62) was established (p<0.001). Normative data for the Portuguese version of Sniffin`Sticks test is provided, showing good validity and reliability and effectively distinguishing patients from healthy controls with high sensitivity and specificity. The Portuguese version of Sniffin`Sticks test identification test is a clinically suitable screening tool in routine outpatient Portuguese settings.

## Introduction

Olfaction is important for our daily living. Impairment of the sense of smell has important consequences for quality of life, health, and safety. Olfaction is considered to be less disabling that other sensory losses, such as blindness and deafness. As a consequence, the medical community considers olfaction as a less important sense and is less studied. Recent advances in our understanding of olfaction changed this. For example, olfactory impairment is an early, frequent and sensitive marker of the preclinical phase of neurodegenerative diseases like Parkinson and Alzheimer diseases. It is present in over 90% of early Parkinson disease stages which, in turn, may lead to early treatment.[1,2]

Current consensus estimates that about 5 percent of the general population suffers from anosmia, and about 20% exhibit hyposmia.[3]

Odor identification is culturally dependent. For example, many smells that are familiar in the USA are not familiar in Europe. Specifically, Portugal has a unique, 800-years old culture which, of course, comes along with many characteristic flavors. Considering this, it is important to establish an olfactory test that distinguishes between normal and pathological situations. In addition, the test, ideally, should be able to monitor changes in olfactory capacity over time, for example, in order to establish the possible effects of treatment.

No validated olfactory test for the Portuguese population currently exists. Thus for a population of 10.5 million people it is impossible to correctly evaluate the sense of smell, make accurate diagnoses, evaluate prognoses, or compare treatment modalities.

The best-validated olfactory tests include the UPSIT (University of Pennsylvania Smell Identification Test),[4] the CCCRC test (Connecticut Chemosensory Clinical Research Center),[5] and the “Sniffin’ Sticks” (SnSt).[6] The latter is a European-designed test, while the first two were developed in North America.

The SnSt has been validated in various countries and populations not only in Europe (e.g Germany and northern European countries [7], Italy [8], Greece [9], and Holland, [10] but also outside Europe like in Australia [11], Sri Lanka [12], Brazil [13] or Taiwan [14].

This work aimed to adapt and validate a reliable Portuguese version of the Sniffin´ Sticks (SnSt) test that would be suitable both for clinical and laboratory settings. Such a test would permit health professionals, industry officials, and others to evaluate the olfactory conditions in Portugal, compare this with the standards of other cultures, and to, diagnose, treat and study multiple related pathologies and conditions.

## Participants and Methods

### Ethics statement

All participants provided informed written consent. The study followed the Declaration of Helsinki 2013 on Biomedical Research Involving Human Participants and was approved by the Ethics Committee of the Faculty of Medicine, University of Coimbra, Portugal.

### Study population

A total of 343 investigations were performed, with 272 participants (203 healthy individuals and 69 hyposmic patients) who were recruited for the normative step of the Portuguese version of SnSt (mean age: 40±17; range: 18–82 years; female/male: 104/99) and 71 of those performed a retest (mean age: 22±3; range: 20–41 years; f/m: 33/38). Participant recruitment took place from September 2013 to March 2015.

After a detailed and structured clinical history, a complete ENT exam was performed, including nasal endoscopy. Cognitive impairment was excluded using the Mini-Mental State Examination.[15] A history of olfactory problems, current upper respiratory tract infection, diabetes, previous nasal surgery, neurological or psychiatric diseases, or major head trauma were exclusion criteria.

### Smell testing

For orthonasal olfactory testing, the SnSt were used (Burghart GmbH, Wedel, Germany).[6,7] This test consists of 3 subtests, namely, tests for odor threshold (T), odor discrimination (D), and odor identification (I). Results of the 3 subtests are typically summed up and presented as a composite TDI score. Normative data is available based on multi-centered European examinations.[7,16]

Odor threshold testing identifies the least detectable concentration of an odorant (phenyl ethyl alcohol) that can be perceived by a participant. It is determined by the administration of 16 geometrically increasing dilutions of the odorant in a single-staircase design within a 3-alternative forced-choice procedure. Three pens are presented starting from the pen with the weakest dilution, with two pens containing the solvent and the third the odorant at a certain dilution. The participant’s task is to identify the odor-containing pen. Each change from weaker to stronger or stronger to weaker is considered a “reversal”. Threshold is defined as the mean of the last four staircase reversal points. Participants’ scores range between 1 (the highest concentration is not perceived) and 16 points (the lowest concentration is perceived).[6,7]

The SnSt odor discrimination test assesses the ability to distinguish a certain odorant from another using a 3 alternative forced choice technique (16 triplets). In this task, no naming or formal identification of the odorant is necessary.

The SnSt odor identification testing involves a multiple forced choice identification of 16 odors from a list of 4 descriptors each. It is known to have a strong cultural connotation and needs to be adapted in order to avoid a situation in which the local population is unfamiliar with the odors presented or with the descriptors used for the multiple choice task.

### Methods

Our protocol design included 4 experiments:

#### Experiment 1 –cultural adaptation of the identification subtest

In order to determine odor familiarities of the Portuguese population, the SnSt identification test was translated into Portuguese by a native English speaker and a native and bilingual Portuguese. Several different descriptors for the same original descriptor were found. Instead of performing a classical two investigator results reconciliation and back translation,[17] all descriptors were paneled for familiarity survey testing. Participants (n = 67) were asked to rate odor descriptors according to how familiar each odor seemed to them, using a Likert-type scale ranging from 0 to 5 (0 = unknown, 5 = highly familiar). Averaged results were converted to a percentage scale and presented in Table 1.

**Table 1:** Survey results for familiarity of odor descriptors after translation to Portuguese language. After using a Likert type scale ranging from 0 to 5 (0 = unknown, 5 = highly familiar), average results are presented in a percentage scale.

Original odor descriptor	Proposed Portuguese descriptor	%	Original odor descriptor	Proposed Portuguese descriptor	%
Garlic	Alho	98%	Walnut	Noz	78%
Glue	Cola	97%	Fir	Pinheiro	78%
Coffee	Café	96%	Peach	Pêssego	77%
Menthol	Mentol	94%	Blackberry	Amora	76%
Fish	Peixe	94%	Pepper	Pimenta	76%
Rose	Rosa	94%	Plum	Ameixa	76%
Onion	Cebola	91%	Rum	Rum	76%
Cheese	Queijo	90%	Gummy candy	Goma de fruta	75%
Peppermint	Hortelã	89%	Pear	Pêra	75%
Cinnamon	Canela	88%	Licorice	Anis	75%
Lemon	Limão	87%	Grass	Relva	74%
Banana	Banana	86%	Chive	Cebolinho	74%
Chocolate	Chocolate	86%	Pineapple	Ananás	74%
Orange	Laranja	86%	Anise	Anis	73%
Strawberry	Morango	86%	Smoke'	Fumo	70%
Vanilla	Baunilha	86%	Coconut	Coco	68%
Spearmint or chewing gum	Pastilha elástica	86%	Ham	Fiambre	68%
Wine	Vinho	86%	Raspberry	Framboesa	68%
Leather	Couro	85%	Sauerkraut	Couve	66%
Carrot	Cenoura	84%	Spearmint or chewing gum	Hortelã-pimenta	66%
Cherry	Cereja	84%	Licorice	Alcaçuz	62%
Turpentine	Diluente de tinta	83%	Grapefruit	Toranja	62%
Cigarette	Fumo de cigarro	82%	Candle smoke	Fumo de vela	62%
Bread	Pão	82%	Fir	Abeto	60%
Apple	Maçã	80%	Clove	Cravinho	56%
Melon	Melão	80%	Turpentine	Terebintina	56%
Chamomile	Camomila	79%	Sauerkraut	Chucrute	42%
Mustard	Mostarda	79%	Gummy candy	Ursinho de goma	33%
Honey	Mel	78%			

The successful identification of individual odorants from a list of four descriptors should be >75% in healthy participants.[6] Accordingly, original answer sheet was modified.

Combined verbal and nonverbal information was provided for all odorants and distractors.

#### Experiment 2 –normative values

The Portuguese version of the Sniffin`Sticks test (SnSt-pt) test was administered to 203 healthy participants (39.1±15.1 years; 104/99 f/m; 25 smokers) with the aim of defining the relevant normative values and the validity of the test in the Portuguese population.

#### Experiment 3 –validity: differentiate normal vs anosmia

A third experiment included a group of 69 patients previously reported as having olfactory loss (40.7±20.6 years (range 20–81)). This group was tested with SnSt-pt in order to examine if the test could discriminate between healthy controls and people indicating olfactory loss. It included people with advanced Parkinson’s disease, nasal polyps and severe septal deviations.

#### Experiment 4 –reliability, test-retest

One last experiment re-evaluated 71 healthy participants with a 1 month interval in order to examine test–retest reliability of the SnSt-pt.

### Statistical methods

Statistical analysis was performed with SPSS version 22 (SPSS Inc., Chicago, IL, USA). Data was summarized using mean ± standard deviations and 95% confidence intervals for continuous variables and percentages for categorical data. Data was examined for normality with the Kolmogorov-Smimov test. SnSt-pt scores were compared using independent sample t tests and one-way analysis of variance (ANOVA) with post hoc Bonferroni tests. Correlational analyses were performed using the Pearson´s Chi-squared test. To assess the factors that independently influence SnSt-pt, multiple linear regression analysis was performed using TDI and T score as the dependent variable and age, gender and current smoking status as covariates. Multiple logistic regression analysis was performed to predict the usefulness of SnSt-pt TDI and T scores to differentiate patients from controls. Test–retest reliability was evaluated by means of the concordance correlation coefficient on 71 randomly selected healthy participants who were re-assessed with the SnSt-pt about 1 month after the first evaluation. Cronbach´s alpha, Pearson’s correlation statistic and intra-class correlation coefficient (ICC) were calculated. Bland–Altman plots showed the agreement between test and retest measurements. The level of significance was set at 0.05.

## Results

The odor identification test required both translation and the replacement of distractors unfamiliar to the Portuguese population. [6] Results were converted into a percentage scale and results displayed in Table 1.

The original answer sheet was modified to include more familiar descriptors. In particular seven names of odors and descriptors were replaced according to the familiarity survey (Table 2).

**Table 2:** Replacement in the names of odors and descriptors.

Original English descriptor (SS)	Portuguese direct translation (familiarity in %)		SnSt-pt Portuguese descriptor adaptation (familiarity in %)		English “retro-translation” of new descriptor
Gummy candy	Ursinho de goma	33%	Goma de fruta	75%	Gummy candy
Sauerkraut	Chucrute	42%	Couve	66%	Cabbage
Turpentine	Terebintina	56%	Diluente de tinta	83%	Paint thinner
Fir tree	Abeto	60%	Pinheiro	78%	Pine tree
Licorice	Alcaçuz	62%	Anis	73%	Anise
Grapefruit	Toranja	62%	Laranja	86%	Orange
Peppermint	Hortelã-pimenta	66%	Hortelã	89%	Spearmint

After English-Portuguese translation (1^st^ column), Portuguese direct translation descriptors with the lowest familiarity scores were replaced for similar (adapted descriptors), presented in the third column (respective English translations in the 4^th^ column).

After replacing the names of odors and descriptors with low familiarity indexes as described in Table 2, an overall improvement in familiarity of 24±9.5% was achieved.

The SnSt-pt test was administered to 203 healthy participants to define normative values and the validity of the test in the Portuguese population (Table 3).

**Table 3:** Sniffin´ Sticks-pt normative data from healthy participants in Portugal.

		TDI	Threshold	Discrimination	Identification
**18–35years (n = 94)**					
Mean ± SD		36.98±3.96	10.83±2.31	12.05±2.16	14.10±1.21
Range		25.25–43.5	4–14.5	6–15	9–16
Percentiles	10	**31.75**	**7.00**	**8.00**	**13.00**
	25	34.50	9.50	11.00	13.00
	75	39.75	12.50	14.00	15.00
	90	41.75	13.50	14.00	15.00
**36–55 years (n = 69)**					
Mean ± SD		34.76±3.58	9.41±3.42	11.81±2.06	13.54±1.30
Range		25.25–41.25	1.75–15.00	6.00–16.00	8.00–16.00
Percentiles	10	29.25	5.50	10.00	13.00
	25	33.00	5.75	11.00	13.00
	75	36.75	12.50	13.00	14.00
	90	40.25	14.25	15.00	15.00
**>55 years (n = 40)**					
Mean ± SD		35.49±5.00	10.59±2.79	11.53±1.93	13.38±2.65
Range		25.25–40.50	4.25–12.75	6.00–15.00	4.00–16.00
Percentiles	10	26.35	5.53	8.10	10.00
	25	32.56	8.31	11.25	12.00
	75	40.25	12.50	13.00	16.00
	90	40.50	12.50	13.00	16.00

TDI: total score (sum of threshold, discrimination, identification scores). SD = standard deviation. Percentile 10 of the 18–35 year old group defines the cutoff point for hyposmia in the population.

**Fig 1:**
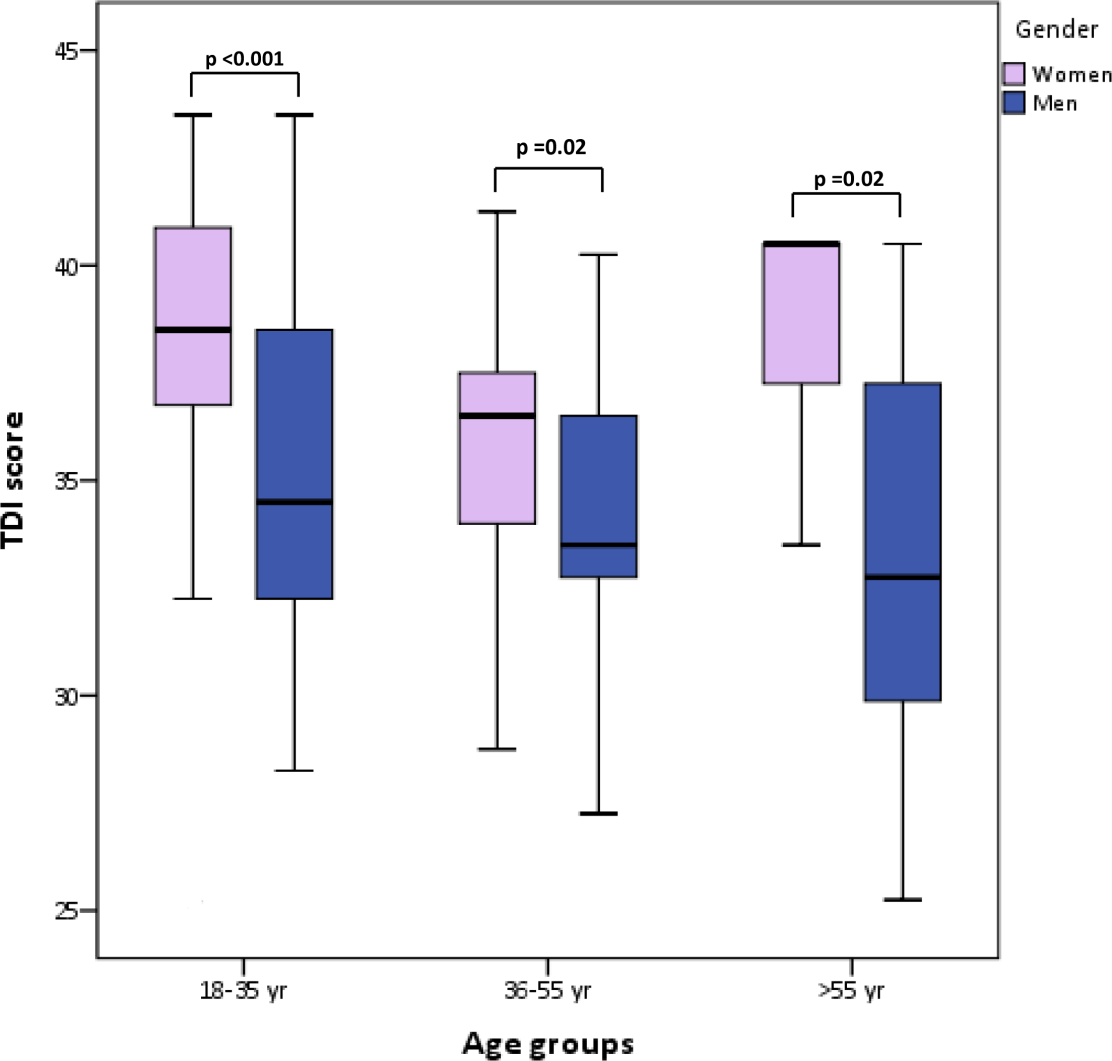
Box plot of TDI score for 3 age groups and for women and men. TDI = composite score of T, D and I. T = threshold; D = discrimination; I = Identification. The median (horizontal line) is shown within each box, the extreme of the “whiskers” indicate the central 95% of each observation. No other significant relations were observed.

After observing its normal distribution, a multiple regression analysis was run to predict the SnSt-pt TDI score as the dependent variable in relation to age, gender and smoking status. Age (r = -0.271, p<0.001), gender (r = -0.399, p<0.001) and smoking status (r = -0.439, p<0.001) were significant independent predictors of the TDI score, F(3,199) = 30.19, p<0.001) and the adjusted R^2^ was 0.30, indicating that these variables explain 30% of the TDI score variation. Data is presented in Fig 1 and Table 4.

**Table 4:** Normative data of TDI discriminated by age and gender. The cutoff point has been defined by the 10^th^ percentile of the distribution of the scores in the age group 18–35 years.

	TDI		Threshold		Discrimination		Identification	
**18–35yr (n = 94)**	Men	Women	Men	Women	Men	Women	Men	Women
10^th^ percentile	29.45	34.35	6.8	9.5	8	10	13	13

TDI = sum of threshold, discrimination and identification scores. Men = 38; women = 56.

**Fig 2:**
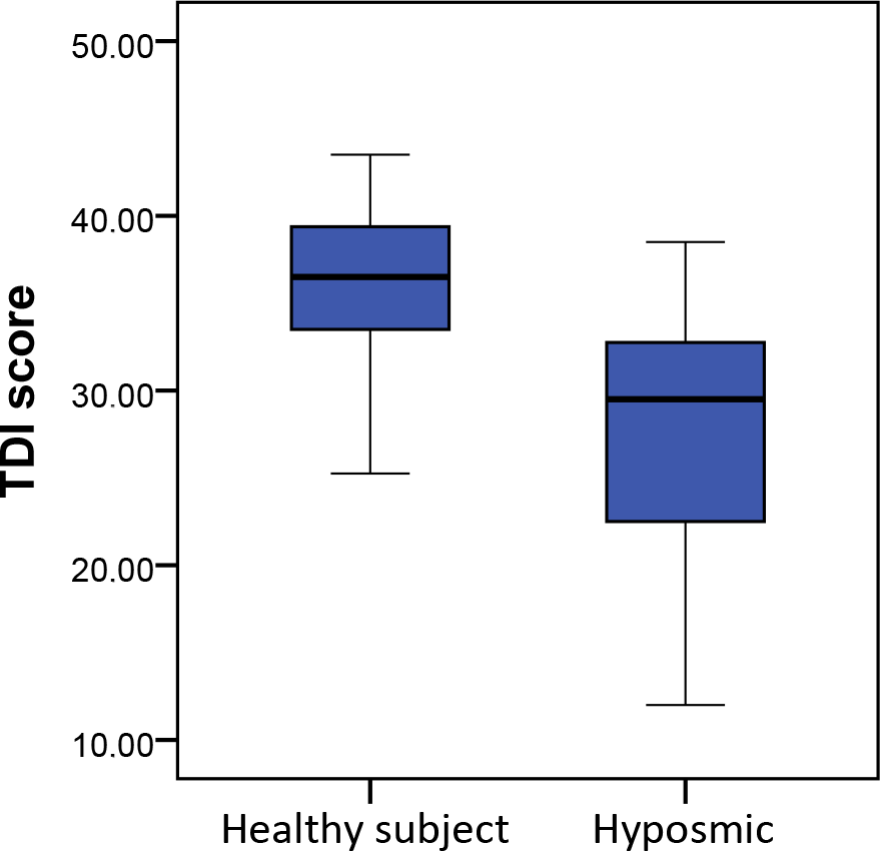
Box plot comparing TDI scores between the healthy participants group and the “hyposmic” control group. TDI = composite score of T, D and I. T = threshold; D = discrimination; I = Identification.

A group of 69 previously known patients with olfactory loss was tested in order to examine whether or not the test is capable of discriminating between healthy controls and patients with an olfactory disorder (Table 5). TDI score was significantly lower in the hyposmic group (27.89±6.48 versus 35.86±4.38, p<0.001) (Fig 2).

**Table 5:** Sniffin´ Sticks Portuguese version results for the hyposmic group.

	Mean	Standard deviation	min	max	Percentiles		
					10	25	75
TDI	27.89	6.47	12.00	28.50	17.25	22.50	32.75
T	8.16	3.00	1.00	13.50	3.25	6.63	10.50
D	8.94	2.18	2.00	13.00	6.00	8.00	10.00
I	10.62	3.20	4.00	15.00	4.00	7.50	13.00

TDI = composite score of T, D and I. T = threshold; D = discrimination; I = Identification.

**Fig 3:**
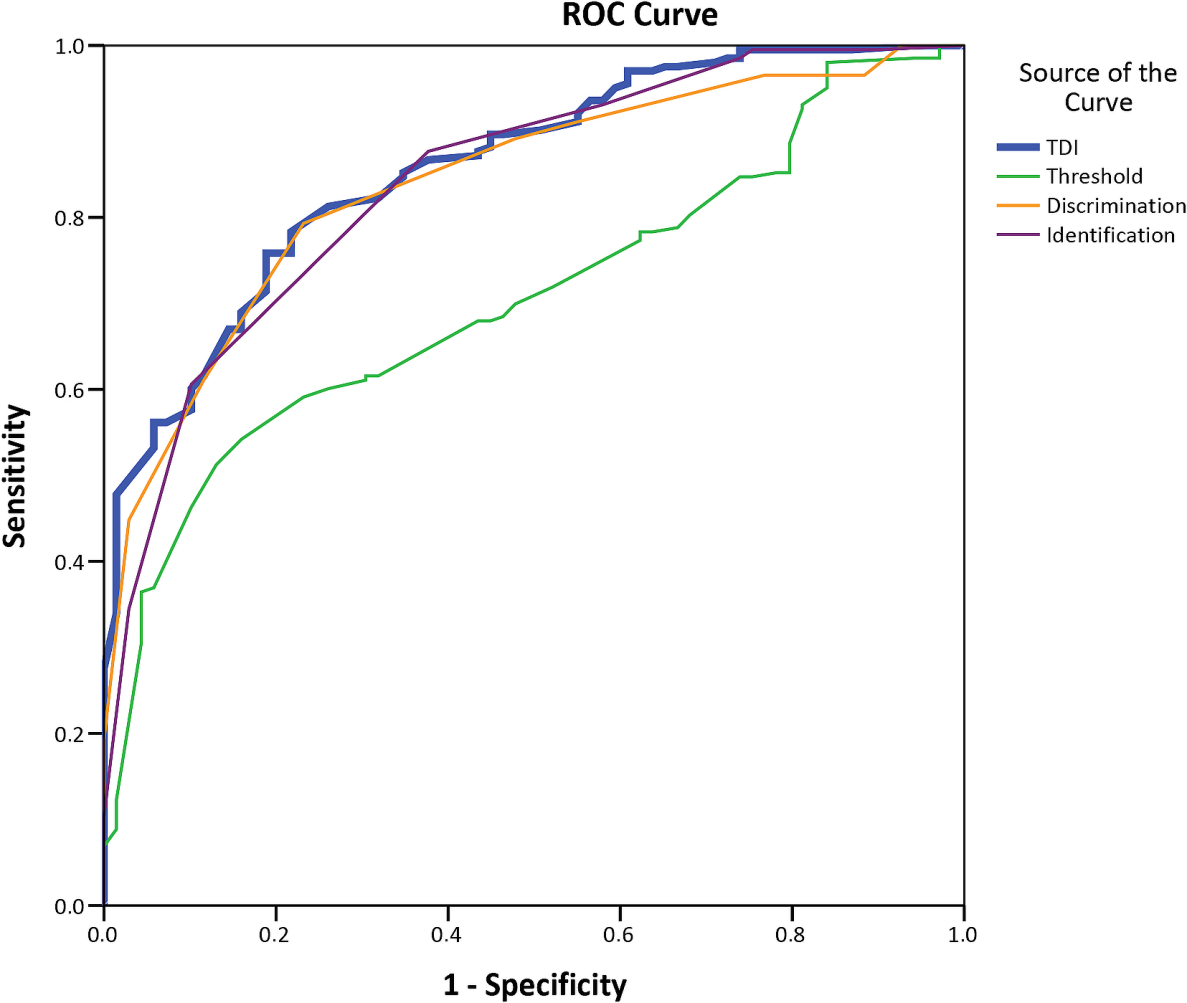
Area under the curve for TDI 0.86 (95%CI 0.81–0.91); Threshold 0.71 (95%CI 0.64–0.77); Discrimination 0.84 (95%CI 0.79–0.89); Identification 0.84 (95%CI 0.79–0.89), p<0.001. TDI = composite score of T, D and I. T = threshold; D = discrimination; I = Identification.

The selected hyposmia reference limits (cut-off value of 31.75) showed a SnSt-pt sensitivity of 78% (95% C.I.: 64.7–87.5) and a specificity of 88% (95% C.I.: 83.8–92.7) with a positive predictive value of 65% and a negative predictive value of 93%. The positive likelihood ratio was 46.92 and the negative 0.25. Receiver operating curve (ROC) analyses confirmed that SnSt-pt is highly accurate (Fig 3).

The TDI test (35.78±4.06) -retest (38.36±3.36) correlation was r_71_ = 0.86 and was found to possess a high degree of internal consistency (Cronbach alpha = 0.91, adjusted R^2^ = 0.73 and ICC single measure = 0.84, 95% C.I. = 0.76–0.90). The strong effect size observed is important in evaluating the magnitude of the reliability: F(37,33) = 6.98, p<0.001. ETA squared = 0.887, standardized response mean difference glass´s Δ = 1.00 and *Cohen* d = 0.85. The area under the curve for SnSt-pt was 0.86 (95%CI 0.81–0.91), p<0.001. (Fig 3)

**Fig 4:**
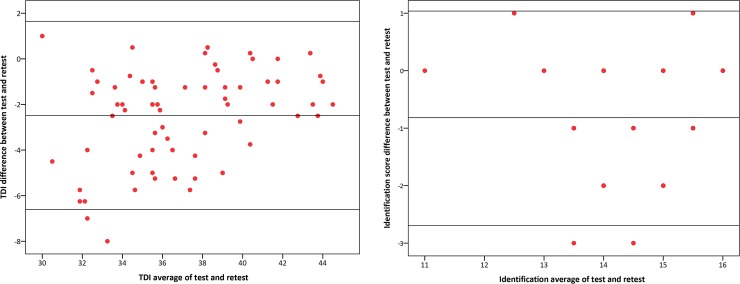
A Bland Altman agreement plot for TDI and Identification scores for test and retest. The middle horizontal line represents the mean, and the upper and lower lines the 95% confidence limits of the mean.

Identification test may be used for screening purposes (Table 6). It shows a test (14.13±1.08) retest (14.94±1–09) correlation of r_71_ = 0.62, p<0.001. It exhibited a high internal consistency (Cronbach alpha = 0.77, adjusted R square = 0.73 and ICC single measure = 0.62, 95% C.I. = 0.46–0.90). Area under the curve was 0.84 (95%CI 0.79–0.89), p<0,001 (Fig 3). A Bland Altman agreement plot for TDI and Identification scores for test and retest is presented in Fig 4.

**Table 6:** Reliability measures of the SnSt-pt subtests. Cronbach alpha and ICC are good at 0.70 and above.

	Cronbach alpha	SnSt-pt correlation (r_71_, p<0.001)	Original Sniffin`Sticks correlation[16]	Adjusted R square	ICC single measure
**TDI**	0.91	0.86	0.72	0.73	0.84, 95% CI = 0.76–0.89
**T**	0.94	0.92	0.61	0.85	0.89, 95% CI = 0.83–0.93
**D**	0.81	0.71	0.54	0.49	0.68, 95% CI = 0.54–0.79
**I**	0.77	0.62	0.73	0.38	0.62, 95% CI = 0.46–0.75

## Discussion

To our knowledge, this work presents the first olfactory test validated in the Portuguese population. Normative data for age and gender is presented. The ability to discriminate healthy participants from persons with impaired olfactory ability is established and showed good reliability. About 10 million people now have access to and can be the subjects of an olfaction test adapted to their own habits and culture. And that is important not only for research but also for clinical purposes. The ability to accurately and reliably assess olfactory function has very important clinical, safety and medico-legal implications.

Olfactory tests are known to have strong cultural affinities.[18,19] We adapted SnSt instead of USPIT or CCCTRC test because of its European cultural background. Additionally, the SnSt permits the study of the three components of olfaction (threshold, discrimination and identification), rather than only identification, as does the UPSIT.[6]

We have attempted to rule out cultural bias factors, which some have suggested to be one of the contributors to the lower rates of identifiability achieved for certain odors in the original test. Nevertheless, it still remains with a contextual and linguistic effect, reduced by the non-verbal descriptor cues and translation design we employed.

Gummy candy, sauerkraut, fir, grapefruit, liquorice, turpentine and peppermint are not commonly seen in Portugal, as is demonstrated by the results of the familiarity survey we performed. Therefore, the names of each of these substances were replaced by more common Portuguese designations, as shown in Table 2. Importantly, these modifications do not require changes in how the test is manufactured.

We chose to use both verbal and nonverbal ways of identifying odors because of the widely different backgrounds and educational status of the subjects in both our clinics and laboratory. Several papers mentioned no difference in using only word alternatives.[6,20] Yet, we chose to include nonverbal approaches as well. We did this because we sought to create a test that would be useful for the elderly, for people with dementia and psychiatric problems, and for the deaf-blind. Moreover, including a nonverbal approach helped reduce total testing time while it made taking the test more interesting for participants.

In our study, the SnSt-pt test was administered 343 times as part of our efforts to develop normative values in relation to different age and gender groups. The 10^th^ percentile of the 18–35 year old group was used as the level at which normosmia could be distinguished from hyposmia (TDI = 31.75; T = 7; D = 8 and I = 13).[7] Original SnSt normative data are applicable mainly to the populations of German-speaking countries. The original data established a TDI of 30.3 as a separation point distinguishing normosmic and hyposmic people.[7] The data from the current study suggest that Portuguese SnSt-pt scores are directly comparable to scores obtained in other countries.[6,8,9,14,21–25], albeit a little higher. This threshold is similar to what has been described for the Greek population.[9] Such findings may be partly explained by the Mediterranean weather, as a warm climate may favor higher thresholds.

Although a complete olfactory workup, including a TDI score, is important for research purposes and for some clinical cases, it typically is too time-consuming for use in a busy clinical setting. Therefore, we have also presented data on the identification score alone. Focusing on this alone permits providers to evaluate olfactory capacity with an approach that takes less time than does the full SnSt battery. The identification test was found to be a clinically suitable screening tool for the Portuguese population. In fact, the current study indicates that the odor identification test is a reliable screening tool with good test-retest reliability (r = 0.62, p<0.001) and an area under the ROC curve of 0.84. These results suggest this approach has value as a way of differentiating between “normosmic” and “hyposmic” people.

The threshold test is usually considered the most sensitive olfactory examination of overall olfactory function[26,27] and seems to accurately reflect peripheral olfactory function.[27] Nevertheless, the threshold test is characterized by considerable within- and across-participant variability in relation to age, gender and smoking status,[7,28] as was confirmed in our test retest study.

In relation to the evaluation of a person’s olfactory capabilities, older age, male gender and active smoking status are each well-known to diminish olfactory capabilities.[3,16] Nevertheless these factors explain no more than about 30% of the variation in TDI score in this study. Accordingly, it seems reasonable to conclude that the TDI score is an independent predictor of hyposmia.

SnSt-pt showed significantly lower scores in hyposmic patients compared to healthy controls, as matched for age and sex (p<0.001). ROC analyses indicated that SnSt-pt distinguishes patients from controls with high sensitivity and specificity, supporting the role of SnSt-pt in detecting hyposmia. In a clinical setting, TDI and T score may be used to identify a hyposmic person.

The SnSt-pt TDI with a r = 0.86 compares well with the reported reliability of both the Sniffin`Sticks test (r = 0.72)[6] and the UPSIT 0.98.[29] Notably, the identification test correlation, r = 0.62, was lower than that observed for the German version of Sniffin`Sticks (r = 0.73).[6,30] Few published studies have evaluated the reliability of suprathreshold tests other than odor identification tests.[31] The reliability of the discrimination subtest is r = 0.71, which is higher than that of the atypical identification subtest but lower than the subtest for threshold. These findings are in line with those of other reports. [29,30]

Familiarity with stimuli, a learning effect and the feedback provided after the initial testing session, may explain the fact that retest scores tended to be higher than initial scores. Notably, the staircase method we used is known to be associated with elevated levels of false positives. When retesting thresholds, we started the test at the values attained during the previous testing session. We followed this approach in order to reduce the length of time required for testing and to lessen the opportunity for false positives to appear in responses.[32]

## Conclusion

The Portuguese cross cultural adaptation of the SnSt test confirms the validity and reliability of SnSt-pt in the Portuguese population. The study provides distinct and integral normative data for each of 3 age groups and for both genders. SnSt-pt distinguishes patients from healthy controls with high sensitivity and specificity. The SnSt odor identification test is a tool suited for the routine clinical workup of patients and for a range of additional uses in healthcare and in industry.
